# Synergic interaction between amyloid precursor protein and neural cell adhesion molecule promotes neurite outgrowth

**DOI:** 10.18632/oncotarget.7348

**Published:** 2016-02-12

**Authors:** Keping Chen, Huixia Lu, Tianli Gao, Xiulei Xue, Chunling Wang, Fengqin Miao

**Affiliations:** ^1^ Zhongda Hospital, School of Medicine, Southeast University, Nanjing 210009, China; ^2^ Key Laboratory of Developmental Genes and Human Disease, Ministry of Education, Institute of Life Science, Southeast University, Nanjing 210009, China

**Keywords:** amyloid precursor protein (APP), neural cell adhesion molecule (NCAM), neurite outgrowth, Alzheimer's disease (AD)

## Abstract

Alzheimer's disease (AD) is one of the most common neurodegenerative diseases worldwide. The main features of AD are the pathological changes of density and distribution of intracellular neurofibrillary tangles (NFT) and extracellular amyloid plaques. The processing of amyloid beta precursor protein (APP) to β-amyloid peptide (Aβ) is one of the critical events in the pathogenesis of AD. In this study, we evaluated the role of the interaction of neural cell adhesion molecule (NCAM) and APP in neurite outgrowth using two different experimental systems: PC12E2 cells and hippocampal neurons that were isolated from wild type, *APP* knock-in and *APP* knock-out mice. PC12E2 cells or hippocampal neurons were co-cultured with NCAM-negative or NCAM-positive fibroblasts L929 cells. We found that APP promoted neurite outgrowth of PC12E2 cells and hippocampal neurons in either the presence or absence of NCAM. Secreted APP can rescue the neurite outgrowth in hippocampal neurons from APP knock-out mice. The interaction of APP and NCAM had synergic effect in promoting neurite outgrowth in both PC12E2 cells and hippocampal neurons. Our results suggested that the interaction of APP with NCAM played an important role in AD development and therefore could be a potential therapeutic target for AD treatment.

## INTRODUCTION

Alzheimer's disease (AD), an age-related neurodegenerative disorder, is considered to be one of the most common neurodegenerative diseases, with ~30 million patients worldwide. AD has become an unavoidable public health problem [1]. The main features of AD are the pathological changes of density and distribution of intracellular neurofibrillary tangles (NFT) and extracellular amyloid plaques. The processing of amyloid beta precursor protein (APP) to β-amyloid peptide (Aβ) is one of the critical events in the pathogenesis of AD. Despite extensive research effort toward understanding the normal function of APP, its physiological role remains poorly defined. Blocking APP expression using antisense oligonucleotides provoke a distinct decrease in axon and dendrite outgrowth in embryonic cortical neurons [2]. APP may play an important role in promoting neurite outgrowth. Understanding the basic biology of APP and its physiological role during development is important for a better comprehension of AD [3].

APP interacts with some extracellular matrix proteins. Reelin, the multidomain extracellular protein, interacted with the extracellular domain of APP, and such interaction promoted neurite development [4]. TAG1 had been identified as the ligand for APP that induced APP processing and inhibited neurogenesis through Fe65 [5]. As a transmembrane cell adhesion molecule (CAM) of the Ig superfamily in *Drosophila*, Fasciclin II (FasII) and APPL formed a biochemical complex *in vivo* and that the ability of FasII to promote new synapse formation required an APPL-dependent transduction cascade [6]. Neural cell adhesion molecule (NCAM), an immunoglobulin superfamily member, plays important roles in neuronal development, regeneration, and synaptic plasticity. Homophilic NCAM binding can induce neurite outgrowth [7]. Therefore NCAM has been linked to human brain disorders, such as AD and schizophrenia. We previously found the selective interaction of APP with some NCAM isoforms. Our previous studies showed the conserved central extracellular domain of APP bound to NCAM-140, but not NCAM-180 [8]. The interaction of NCAM-140 with APP may increase phosphorylation levels of ERK1 and ERK2, and may involve in MAPK signaling pathway, which indicated that the interaction potentially play a role in neurite outgrowth and neural development. In this study, we investigated whether the interaction of NCAM and APP can modulate neurite outgrowth and neurogenesis.

Cell cultures of primary neurons or neuronal cell lines are commonly used to study compounds regulating differentiation and plasticity of neuronal processes [9]. In this study, we evaluated the role of the interaction of NCAM with APP in neural development using two different co-culture systems. The first system employed the PC12E2 cells seeded on top of confluent monolayer of NCAM-negative or NCAM-positive fibroblasts L929 cells. The second system used primary hippocampal neurons isolated from wild type, *APP* knock-in, and *APP* knock-out mice.

## RESULTS

### Overexpression of APP in PC12E2 cells promoted neurite outgrowth in either the presence or absence of homophilic NCAM binding

PC12 cells were commonly used in the study of neural differentiation including neurite outgrowth. As a subclone of PC12, PC12E2 is well characterized with its NCAM-induced neuronal differentiation [10]. We therefore used PC12E2 cells to study whether APP-NCAM interaction could contribute to NCAM-induced neurite outgrowth.

**Figure 1:**
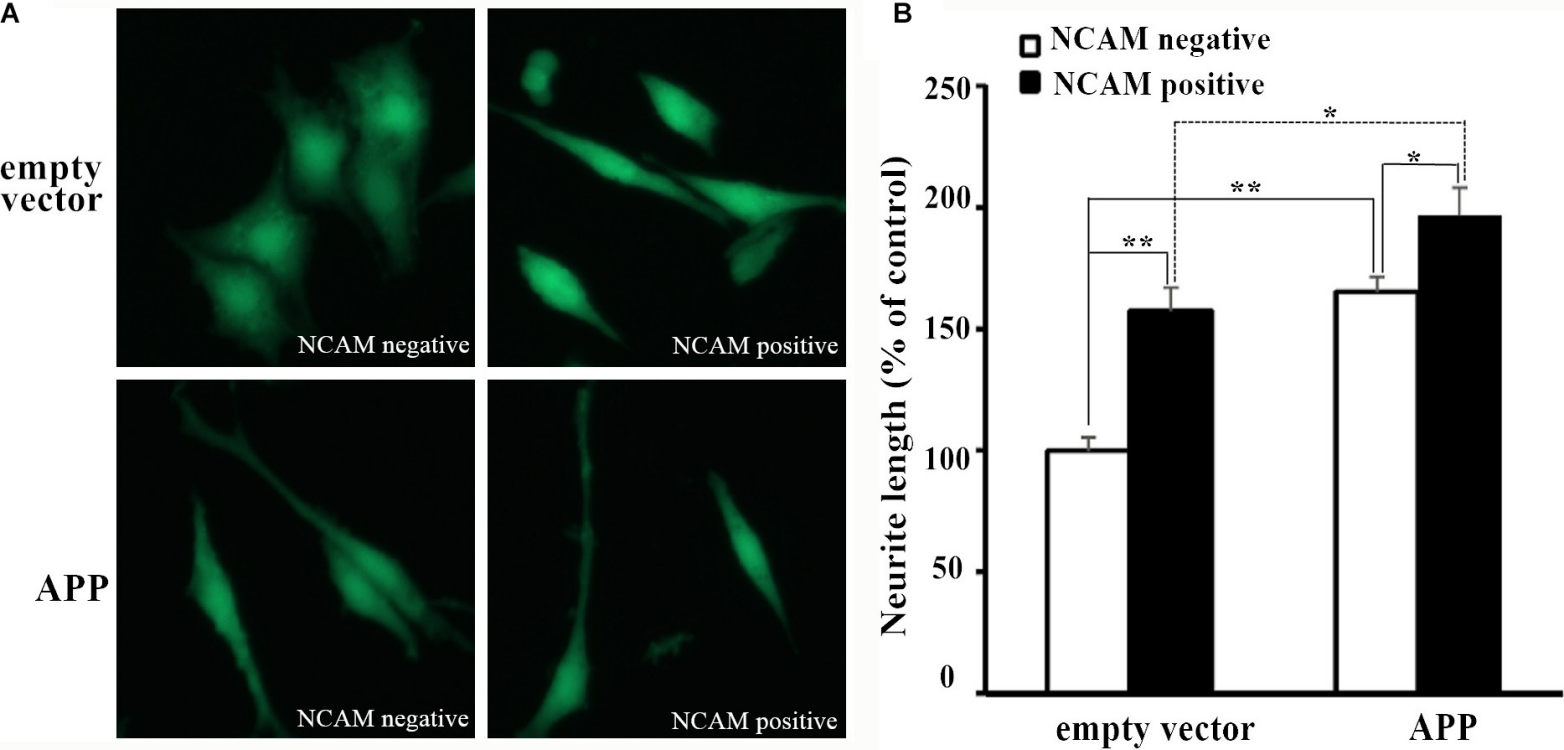
Neurite outgrowth of PC12E2 cells grown on monolayer of fibroblasts L929 (**A**) Confocal images of PC12E2 cells transiently transfected with empty vector pCMV5 (upper panel) or pCMV5-APP (lower panel) and grown on monolayer of NCAM-negative fibroblasts (left panel) or NCAM-positive fibroblasts (right panel). (**B**) Quantification of the neurite length of PC12E2 cells. Neurite length of PC12E2 transfected with empty vector and grown on NCAM-negative fibroblasts was set to 100%. *p < 0.05, **p < 0.01.

PC12E2 cells were seeded on monolayers of NCAM-negative or NCAM-positive fibroblasts L929 cells. As it can be seen from Figure 1A, PC12E2 cells extended long neurite when grown on NCAM-positive fibroblasts while few neurite was seen in cells grown on NCAM-negative fibroblasts. These results indicated homophilic NCAM binding can induce neurite outgrowth. Similar finding was reported by researchers in a previous study [9]. To determine whether APP-NCAM interaction can further promote neurite outgrowth, PC12E2 cells that transfected with either an empty vector (pCMV5) or a vector containing APP (pCMV5-APP) were seeded on monolayers of NCAM-negative or NCAM-positive fibroblasts L929 cells. PC12E2 cells that transfected with pCMV5-APP extended longer neurites in PC12E2 cells grown on NCAM-positive fibroblasts than that on NCAM-negative fibroblasts. The length of neurites was longer in PC12E2 cells transfected with pCMV5-APP than that with the empty vector grown on NCAM-positive fibroblasts (Figure 1A).

Quantifications of the length of neurite outgrowth in PC12E2 cells with or without pCMV5-APP transfection, or grown in NCAM-positive or NCAM-negative fibroblasts were shown in Figure 1B. It can be seen that both APP expression and homophilic NCAM binding promoted neurite outgrowth in PC12E2 cells. Importantly, when PC12E2 cells that transfected with pCMV5-APP were seeded on monolayers of NCAM-positive fibroblasts L929, PC12E2 showed the most neurite outgrowth, which indicated the interaction of APP with NCAM was important in promoting neurite outgrowth in PC12E2 cells.

### APP potentiated neurite outgrowth of hippocampal neurons both in the presence or absence of homophilic NCAM binding

**Figure 2:**
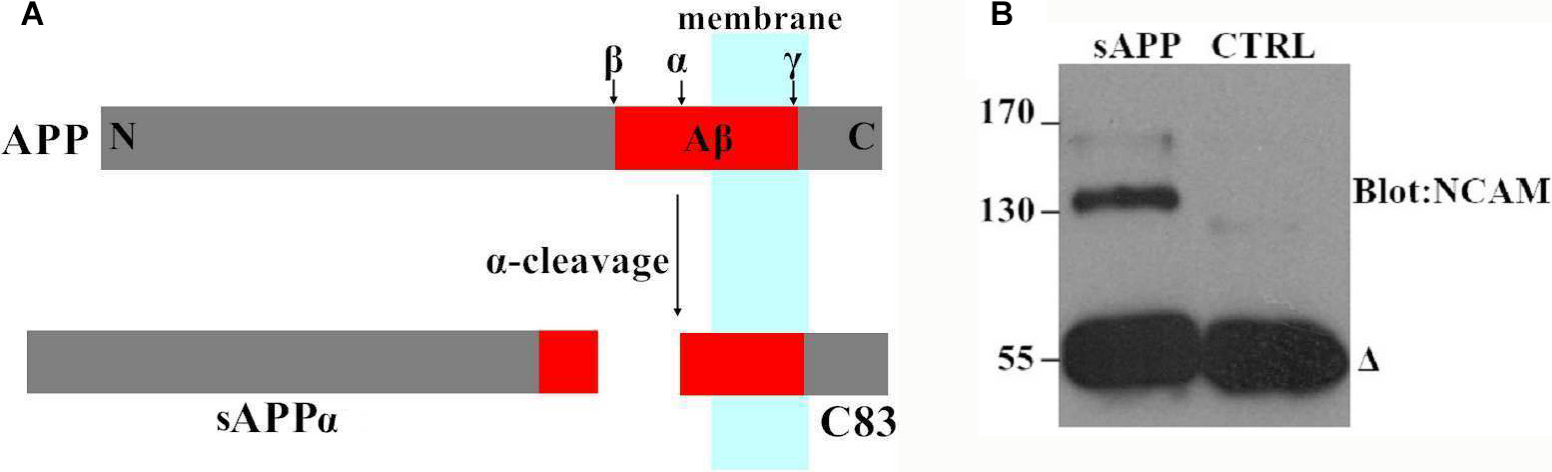
Secreted APP interacted with NCAM-140 in COS7 cells (**A**) Schematic diagram of APP and sAPP. α-secretase cleaves APP to generate the secreted ectodomain (sAPPα) and membrane bound fragment (C83). (**B**) The COS7 cells were transfected with NCAM-140 and maintained in the medium containing secreted APP; cell lysates were immunoprecipitated with 6E10 antibody against secreted APP (sAPP) and probed with an anti-NCAM-140 antibody. As a negative control, an irrelevant antibody was included in the experiment (CTRL). The *delta* denoted antibody heavy chains.

**Figure 3:**
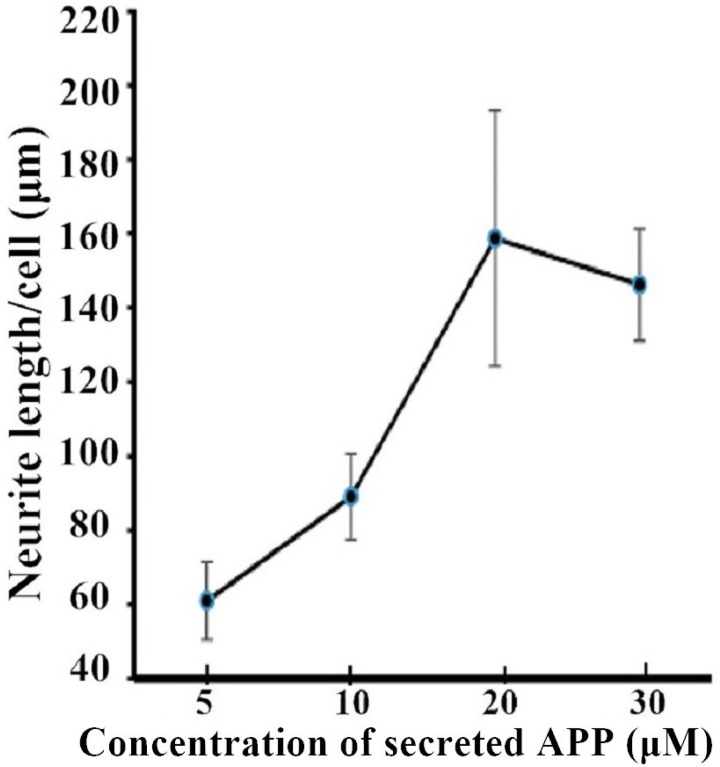
Neurite outgrowth from hippocampal neurons potentiated by secreted APP Neurite length per cell in hippocampal neurons grown in the presence of the indicated concentration of secreted APP for 24 h measured by tracing method. Neurite length presented as mean ± SEM.

**Figure 4:**
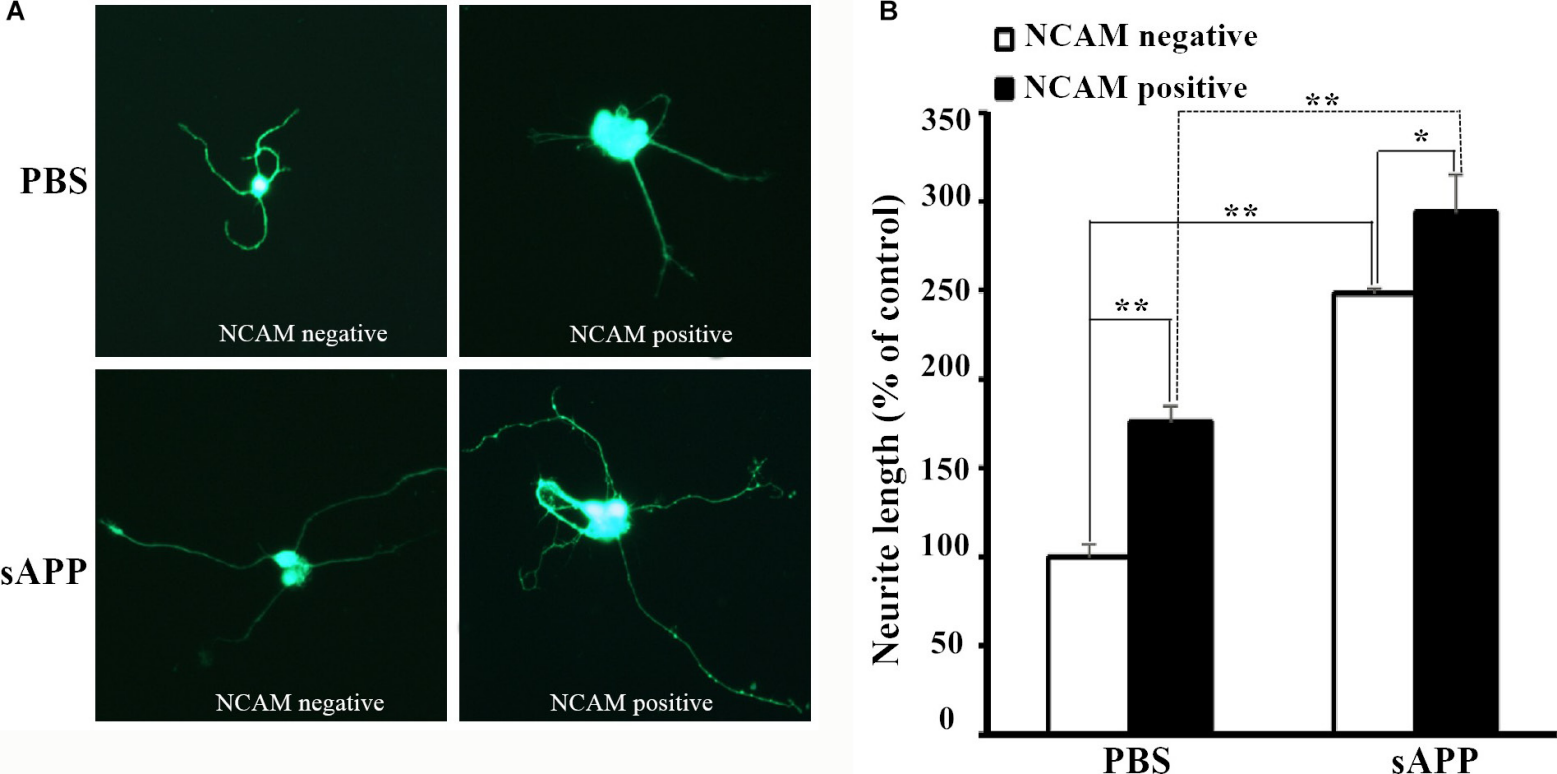
Secreted APP and NCAM potentiated neurite outgrowth (**A**) Confocal images of hippocampal neuron seeded on monolayer of NCAM-negative fibroblasts (left panel) or NCAM-positive fibroblasts (right panel) and maintained in the medium containing secreted APP (lower panel) or PBS (upper panel) as the control. (**B**) Quantification of the neurite length in response to the treatment of secreted APP and NCAM on neurite outgrowth. Neurite length of hippocampal neurons grown on NCAM-negative fibroblasts L929, and maintained in medium containing PBS was set to 100%. *p < 0.05, **p < 0.01.

Next, we investigated the effect of APP on NCAM-induced neurite outgrowth in hippocampal neurons. There was a report by Ring et al. who found that APP C terminus was dispensable and that secreted APP was sufficient to mediate the physiological functions of APP [11]. We also found that secreted APP was able to interact with NCAM-140 similar to full length APP (Figure 2). Therefore we used secreted APP to investigate the role of APP on neurite outgrowth. A stimulation of neurite outgrowth in hippocampal neurons was observed when secreted APP was applied at concentrations of 5–30 μM with a maximal effect at the concentration of 20 μM (Figure 3). In addition, we seeded hippocampal neurons from wild-type mice on monolayers of NCAM-negative or NCAM-positive fibroblast L929 cells and found longer neurite outgrowth in neurons seeded on monolayers of NCAM-positive fibroblasts, compared to neurons on monolayers of NCAM-negative fibroblasts. When the medium was supplemented with 20 μM secreted APP, an increased neurite outgrowth was observed in neurons seeded on monolayers of NCAM-positive fibroblasts, compared to neurons grown on monolayers of NCAM-negative fibroblasts, similar to that in medium containing PBS (Figure 4A). The effect of secreted APP on NCAM-induced neurite outgrowth in hippocampal neuron was quantified and showed in Figure 4B). Similar to PC12E2 cells, both secreted APP and homophilic NCAM binding potentiated neurite outgrowth in hippocampal neurons. Moreover, the effects were synergistic. That is, interaction of APP with NCAM further promoted neurite outgrowth.

### APP and NCAM modulate neurogenesis in hippocampal neurons from *APP* knock-out and *APP* knock-in mice

To further confirm the observed effects of APP and NCAM interaction on neurite outgrowth, we investigated the role of the interaction in neurite outgrowth using hippocampal neurons from wild type, *APP* knock-out and *APP* knock-in mice.

**Figure 5:**
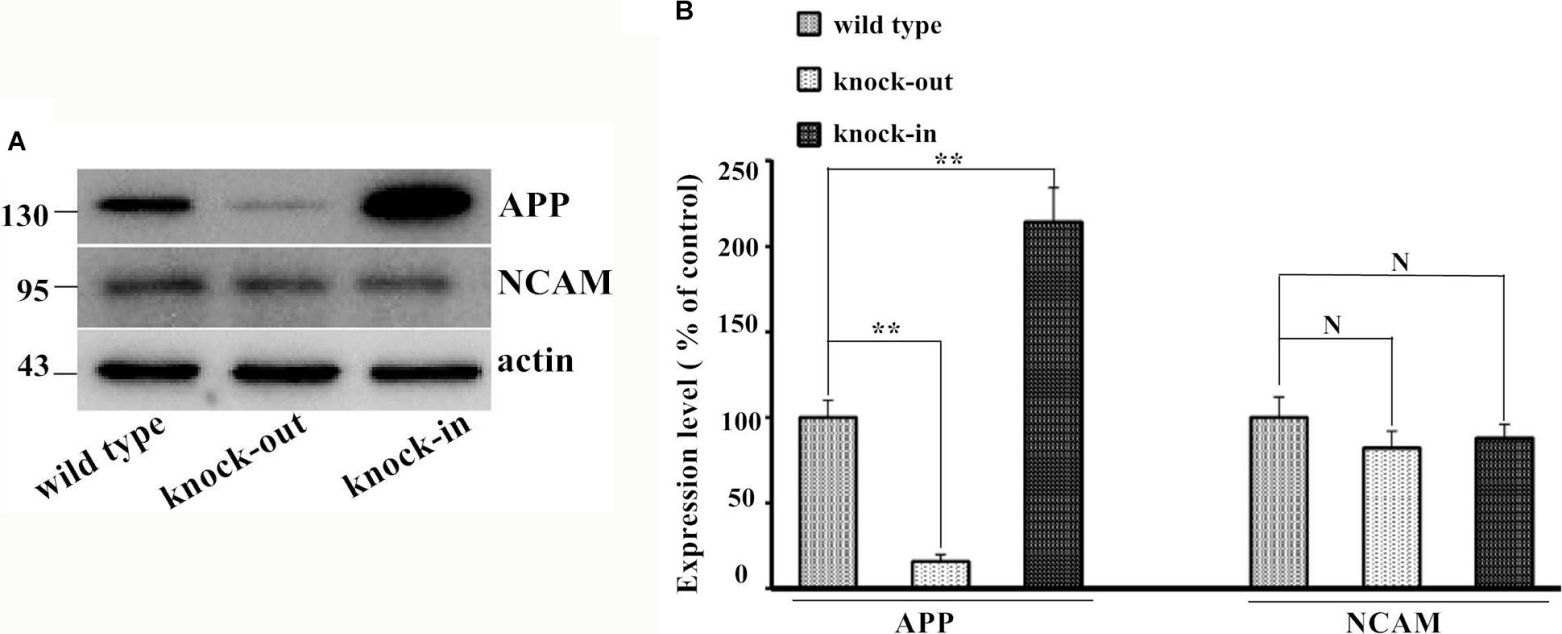
Expression of endogenous APP and NCAM in wild type, *APP* knock-out, and *APP* knock-in mice (**A**) Western blot of the expression of APP and NCAM. (**B**) The quantification of the expression level of each protein. The expression of APP and NCAM was set to 100%. *p < 0.05, **p < 0.01.

**Figure 6:**
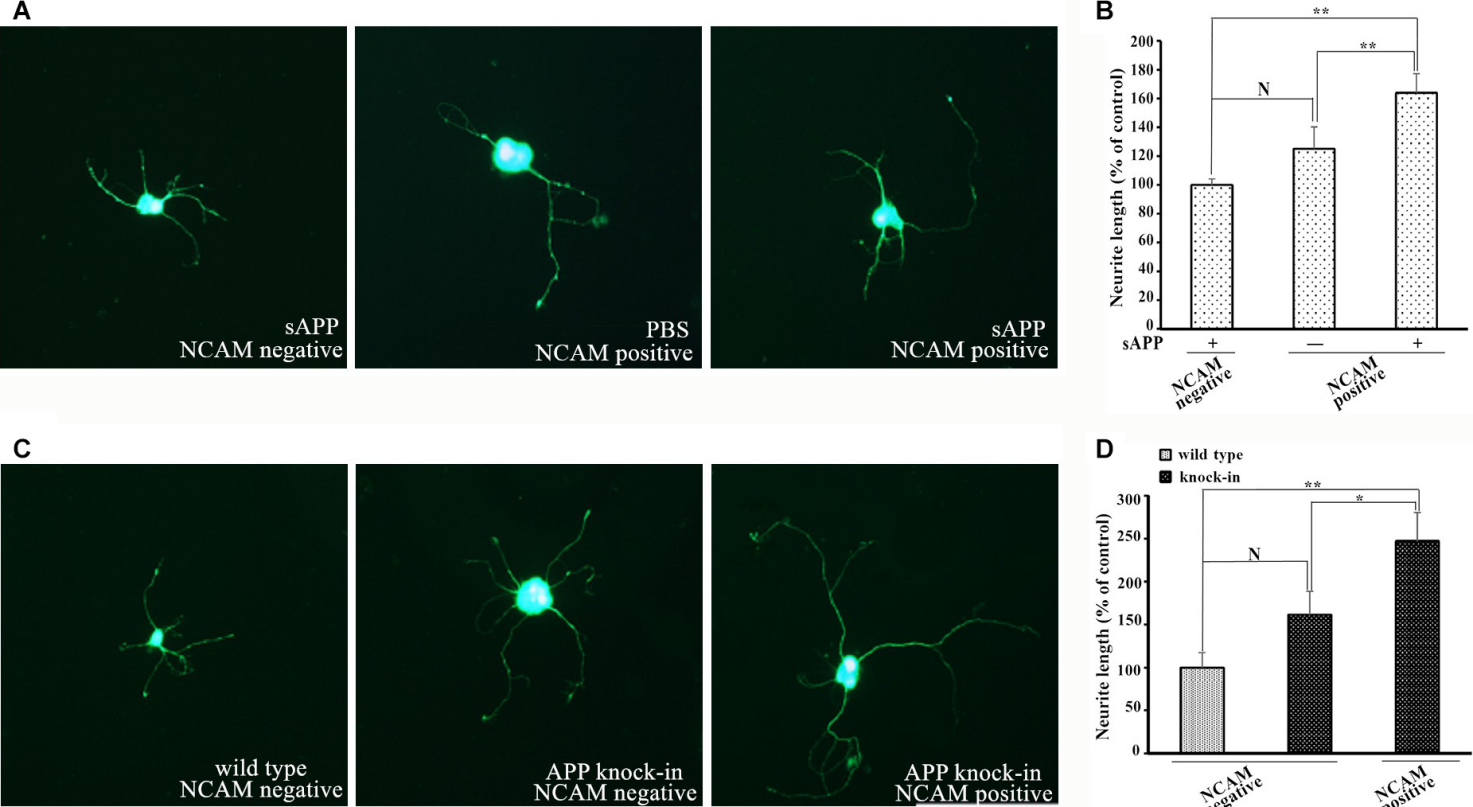
Neurite outgrowth in hippocampal neurons from *APP* knock-out and *APP* knock-in mice (**A**) Confocal images of hippocampal neurons from *APP* knock-out mice grown on monolayer of NCAM-negative fibroblasts in the presence of secreted APP (left), and on monolayer of NCAM-positive fibroblasts in the absence of secreted APP (middle) or in the presence of secreted APP (right) in medium. (**B**) Quantification of neurite outgrowth from *APP* knock-out mice. Neurite length of hippocampal neurons grown on monolayer of NCAM-negative fibroblasts was set to 100%. (**C**) Confocal images of hippocampal neurons from *APP* knock-in mice grown on monolayer of NCAM-negative (middle) or NCAM-positive fibroblasts (right). Hippocampal neurons from wild type mice grown on monolayer of NCAM-negative fibroblasts (left) as the control. (**D**) Quantification of neurite outgrowth. Neurite length of hippocampal neurons from wild type mice grown on NCAM-negative fibroblasts L929 was set to be 100%. *p < 0.05, **p < 0.01.

Figure 5 showed the endogenous expression of APP and NCAM in primary hippocampal neurons from wild type, *APP* knock-out and *APP* knock-in mice. To investigate the effect of APP in neurite outgrowth, isolated hippocampal neurons from *APP* knock-out mice were seeded on monolayer of NCAM-positive or NCAM-negative fibroblasts L929 cells and maintained in medium in the presence or absence of secreted APP. Seven days after the *in vitro* differentiation, hippocampal neurons were stained with microtubule-associated protein 2 (MAP2) antibody to visualize the neurite outgrowth. Neurons seeded on monolayer of NCAM-positive fibroblasts showed increased neurite outgrowth in the presence of secreted APP in medium, compared to that of PBS in the medium. These results indicated that secreted APP can rescue the neurite outgrowth of neurons of knock-out mice (Figure 6A, Figure 6B). In addition, neurons from knock-out mice seeded on monolayer of NCAM-positive fibroblasts showed significant neurite outgrowth, compared to that on monolayer of NCAM-negative fibroblasts (Figure 6A and 6B). On the other hand, hippocampal neurons were isolated from APP knock-in mice and seeded on monolayers of NCAM-negative and NCAM-positive fibroblasts L929 cells. A significant increased neurite outgrowth were observed in hippocampal neuron from APP knock-in mice maintained on NCAM-positive fibroblasts, compared with that on monolayer of NCAM-negative fibroblasts and neurons from wild type mice (Figure 6C and 6D). These results indicated that either APP or NCAM can promote neurite outgrowth, and further confirm that the effect of APP and NCAM interaction was synergistic. The synergic effect of APP and NCAM interaction was similar in both PC2E12 cells and hippocampal neurons.

**Figure 7:**
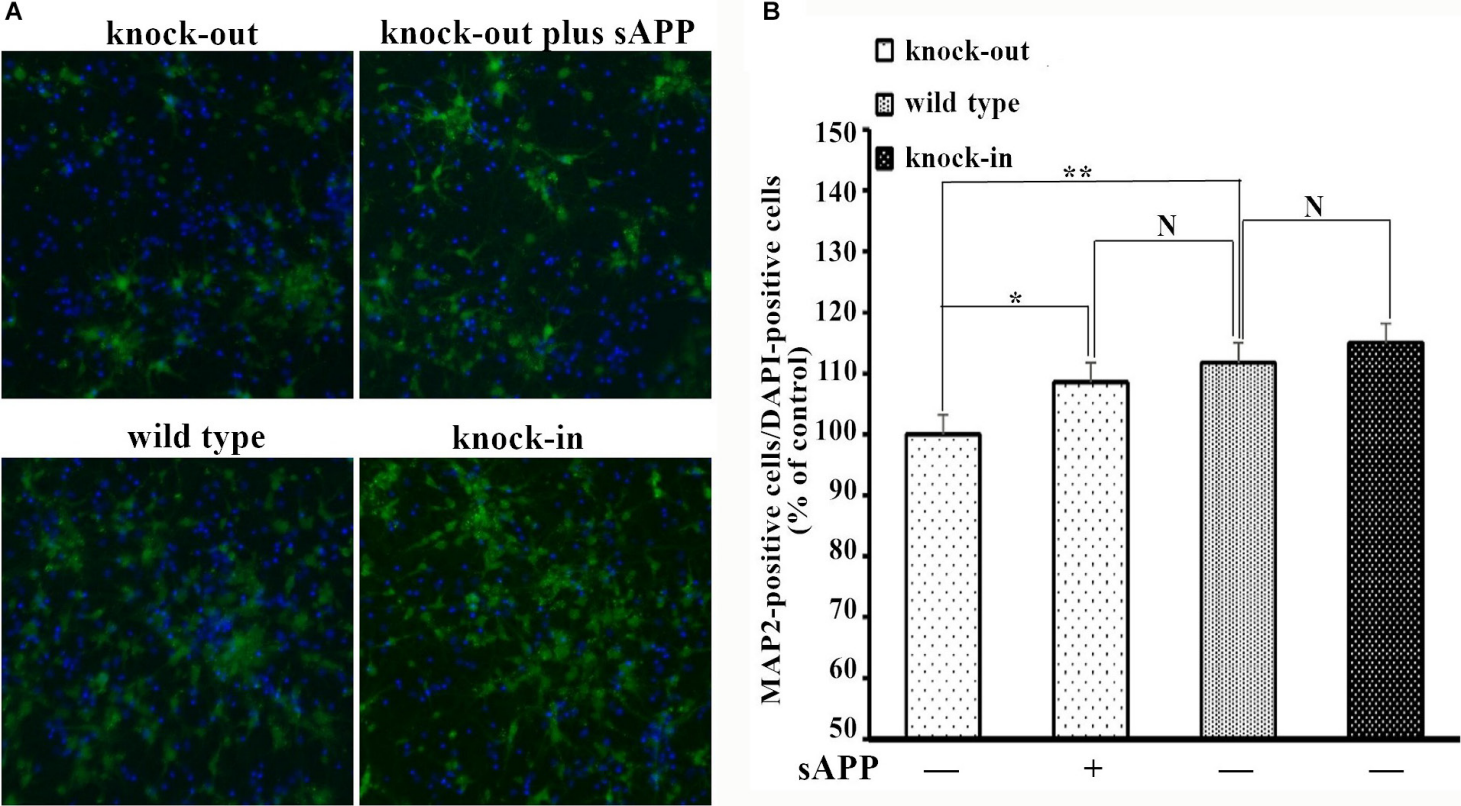
MAP2-positive cells of the hippocampal neurons isolated from wild type, *APP* knock-out and *APP* knock-in mice (**A**) Confocal images of double-stained hippocampal neurons from different mice. (**B**) The numbers of MAP2-positive cells were counted and expressed as a percentage of the number of DAPI-positive cells, and normalized to that of the knock-out mice. The number percentage of MAP2-positive cells of hippocampal neurons from knock-out mice was set to be 100%. *p < 0.05, **p < 0.01.

To further assess the role of the APP-NCAM interaction in neurogenesis, we also studied the number of the hippocampal neurons cultured *in vitro*. Hippocampal neurons were isolated from *APP* knock-out and *APP* knock-in mice and seeded directly in culture plastic dishes. The neurons were double-stained with MAP2 (green) and the nuclear stain DAPI (blue) which represented the total number of cells. The number of MAP2-positive cells was significantly higher in *APP* knock-in mice, compared to *APP* knock-out mice after 7 days in *in vitro* differentiation. Moreover secreted APP in medium can rescue the MAP2-positive cells of the hippocampal neurons from *APP* knock-out mice (Figure 7A and 7B).

## DISCUSSION

The present study was designed to evaluate whether the interaction of APP and NCAM promotes neurite outgrowth in two differential experimental systems, PC12E2 cells and hippocampal neurons. In the PC12E2 cells, the interaction of APP and NCAM significantly promoted neurite outgrowth. There was baseline expression of APP and NCAM in hippocampal neurons of wild type mice. Such endogenous expression is essential to maintain the basic growth of hippocampal neurons.

It was reported that neurite outgrowth from various types of primary neurons was stimulated by homophilic NCAM [12]. Our present study confirmed that neurite outgrowth was promoted when hippocampal neurons and PC12E2 cells were co-cultured with cells expressing NCAM. It is also reported that APP is involved in neurite outgrowth and synaptic function. Numerous *in vitro* studies have established that APP (or secreted APP) plays an important role in neurite outgrowth, including neurite length and branching [13–15]. Our current results from two different experimental systems were in consistent with these previous findings.

Hippocampal neurons from *APP* knock-out mice seeded on monolayer of NCAM-positive fibroblasts and maintained in the presence of secreted APP in the medium, or hippocampal neuron from *APP* knock-in mice grown on the top of NCAM-positive fibroblasts, showed significant increased neurite outgrowth capacity compared with that of hippocampal neurons grown on the top of NCAM-negative fibroblasts. There were more MAP2-positive neurons in *APP* knock-in mice than that in *APP* knock-out mice. The endogenous APP and NCAM can maintain a certain number of MAP2-positive cells in hippocampal neurons, and secreted APP in the medium can increased the number of MAP2-positive cells in hippocampal neurons from knock-out mice.

Both APP and NCAM could modulate MAPK signaling pathway [12, 16]. In our previous studies, an increased ERK1 and ERK2 phosphorylation was found when COS7 cells were co-transfected with APP and NCAM-140. MAPKs signaling pathway including ERKs played an important role in neurite outgrowth [17–18]. In the present study, we found that APP and NCAM interaction can improve neurite outgrowth and increased MAP2/DAPI percentage in hippocampal neurons. It was known that neurite outgrowth is induced by increased phosphorylation of ERK1 and ERK2 in MAPK signaling pathway. The MAPK activation is increased in the AD brain and that activated MAPKs can participate in the abnormal hyperphosphorylation of tau in AD. These studies indicated that the interaction of APP and NCAM synergically promoted neurite outgrowth and play an important role in AD development. Therefore, regulation of APP and NCAM interaction could be a potential therapeutic target for AD treatment. Further study is needed to understand the details of the mechanism by which the interaction of APP and NCAM promote neurogenesis.

## MATERIALS AND METHODS

### Reagents

The following antibodies were used: the 6E10 monoclonal antibody (Covance, Princeton, NJ) identifying amyloid precursor protein (APP). The AG1 monoclonal antibody against the cytoplasmic portions of NCAM (Developmental Studies Hybridoma Bank, DSHB, IA). Rabbit anti-microtubule-associated protein 2 (MAP2) (Chemicon, Rolling Meadows, IL) identifying microtubule-associated protein. DAPI (Roche, Basel, Switzerland) binding to the minor groove of double-stranded DNA was used as a nuclear staining. Secreted APP was purchased from Sigma-Aldrich (Saint Louis, MO).

### Complimentary DNA constructs

The DNA constructs encoding human NCAM-140 in the human expression vector pHb-Apr-1-neo (also termed T4) and the empty vector T4 were generous gifts from Dr. Irina Korshunova (University of Copenhagen, Copenhagen, Denmark) [7, 19]. The NCAM cDNA did not contain exon VASE or exons a, b, c, and AAG. A recombinant vector encoding APP (pCMV5-APP) was prepared as previously described [8]. The correct insertion and expression of recombinant DNA were confirmed by DNA sequencing and Western blotting.

### Co-immunoprecipitation

Transfected COS7 cells with recombinant vector encoding human NCAM-140 were maintained in medium containing secreted APP for 12 h. COS7 cells were lysed with RIPA buffer (25 mM Tris–HCl, pH 8.0, 150 mM NaCl, 1% Nonidet P-40, and 0.1% sodium dodecyl sulfate (SDS)) containing protease inhibitor cocktail tablets (Roche, Basel, Switzerland). The lysates were incubated with the anti-APP antibody (6E10) overnight at 4°C and then the next day incubated with Dynabeads protein G (Invitrogen, Shanghai, China) for 2 h. After washed three times with cold PBS, the beads were resuspended in SDS sample buffer and boiled for 10 min. The samples were separated by SDS-PAGE on 8–10% polyacrylamide gels, and transferred onto a polyvinylidene difluoride (PVDF) membrane (Millipore, Darmstadt, Germany) before blocking with 5% nonfat dried milk in TBST. Membranes were immuoblotted with monoclonal antibody of APP and NCAM, and visualized by SuperSignal West Pico/Femto trial Kit (Thermo Fisher, Rockford, IL) using horseradish peroxidase-conjugated anti-goat IgG.

### Co-cultures of PC12E2 cells with NCAM-negative and NCAM-positive fibroblasts L929 cells

The PC12E2 cells were grown in DMEM supplemented with 5% fetal calf serum (FCS), 10% horse serum (HS), 100 U/ml penicillin, and 100 μg/ml streptomycin at 37°C in a humidified atmosphere containing 5% CO_2_. PC12-E2 cells were grown for 24 h to be transfected with pCMV5-APP encoding the APP or vector alone. All the transfections were carried out using X-tremeGENE HP DNA transfection reagent according to the manufacturer's protocol (Roche, Basel, Switzerland). 24 h after transfection, transfected PC12E2 cells were seeded on top of confluent monolayers of fibroblasts L929 cells. The fibroblasts L929 cells were stably transfected with the vector T4 containing a full-length cDNA encoding human NCAM-140 (NCAM-positive fibroblasts L929 cells) or the vector alone (NCAM-negative fibroblasts L929 cells). These cells were routinely grown in DMEM supplemented with 10% FCS, 100 U/ml penicillin, and 100 μg/ml streptomycin, in a humidified atmosphere at 37°C with 5% CO_2_.

### Primary cultures of hippocampal neurons

The *APP* knock-out mice and *APP* knock-in mice were purchased from NRCMM (the National Resource Center for Mutant Mice, NRCMM, Nanjing, China). Hippocampal neurons were prepared from mice embryos at embryonic day 17 (E17) to E19 according to Maar et al. [20]. Briefly, hippocampi were dissected out in cold PBS solution. After removal of meninges and medulla, the tissues were trypsinized at room temperature for 10 min. Finally, the tissues were mixed with serum to stop the digestion, and then filtered with 400 mesh sieve to collect primary neurons. Cells were grown at 37°C with 5% CO_2_ in Neurobasal medium containing glutamate, L-glutamine, B-27 supplement, 100 U/ml penicillin, and 100 μg/ml streptomycin (Life Technologies, Grand Island, NY).

### Co-culture systems

Monolayers of NCAM-negative and NCAM-positive fibroblasts L929 cells were established by seeding cells in a density of approximately 40000 cells/cm^2^ and maintained for approximately 24 h before establishing co-cultures [9]. PC12E2 cells were seeded at a density of approximately 20000 cells/cm^2^ on monolayers of NCAM-negative or NCAM-positive fibroblasts L929 cells. Co-cultures were grown for 24 h in DMEM supplemented with FCS, HS, penicillin, and streptomycin. The dissociated hippocampal neurons were seeded at a density of approximately 6000 cells/cm^2^ on the top of confluent monolayers of NCAM-negative or NCAM-positive fibroblasts L929 cells. Co-cultures were maintained for 7 days in neurobasal medium supplemented with B27, penicillin, streptomycin, and BSA.

### Neurite outgrowth staining

Co-cultures were fixed in 4% (w/v) paraformaldehyde at 4°C overnight. After washing with PBS containing 1% Trixon-100, cells were blocked with 10% BSA for 1 h. Cells were immunostained with rabbit anti-MAP2 antibody at room temperature for 2 h, and Alexa Fluor secondary antibody for 1 h in an opaque box. Images of cells were obtained in each experiment by computer-assisted fluorescence microscopy using an inverted microscope. The neurite length of hippocampal neurons and PC12E2 cells were estimated using the conventional tracing method [21]. The results were given as mean ± SEM calculated from 3 to 5 independent experiments. Asterisks indicate the statistical significance of *p < 0.05 or **p < 0.01 as compared with control.
